# A PLGA-reinforced PEG *in situ* gel formulation for improved sustainability of hypoglycaemic activity of glimepiride in streptozotocin-induced diabetic rats

**DOI:** 10.1038/s41598-017-16728-0

**Published:** 2017-11-27

**Authors:** Osama A. A. Ahmed, Khalid M. El-Say, Abdulrahman M. Alahdal

**Affiliations:** 1 Department of Pharmaceutics, Faculty of Pharmacy, King Abdulaziz University, Jeddah, Saudi Arabia; 2 Department of Pharmaceutics and Industrial Pharmacy, Faculty of Pharmacy, Minia University, Minia, Egypt; 3 Department of Pharmaceutics and Industrial Pharmacy, Faculty of Pharmacy, Al-Azhar University, Cairo, Egypt; 4 Department of Clinical Pharmacy, Faculty of Pharmacy, King Abdulaziz University, Jeddah, Saudi Arabia

## Abstract

Glimepiride (GMD) is a third-generation sulfonylurea derivative and one of the top three most-prescribed oral antidiabetic drugs. The need for a depot formulation exists, and a safe and effective antidiabetic therapy is the goal of this study. The aims were to design a depot *in situ* gel (ISG) formulation and investigate the main factors that control the initial burst and sustain the GMD effect using the Box-Behnken design. The studied factors were polymer percent (X_1_), plasticizer percent (X_2_) and benzyl benzoate percent in N-methyl-2-pyrrolidone (X_3_). The results revealed that X_2_ is the only factor that showed significant effects on all investigated responses. Scanning electron microscopy images showed that an increase in PEG % improved the smoothness and reduced the porosity of the ISG formulation surface. The GMD plasma levels in diabetic rats revealed no significant difference (p < 0.05) between the maximum GMD plasma concentrations of the optimized GMD-ISG formula (10 mg/ kg) and oral marketed GMD tablets (1 mg/kg). This result ensures that the optimized formula does not exceed the maximum safe plasma concentration. In addition, the optimized GMD-ISG formulation showed a depot effect that lasted for 14 days post-injection. This approach to controlling GMD release using an *in situ* forming system could be useful for improving patient compliance and diabetes treatment effectiveness.

## Introduction

The antidiabetic drug glimepiride (GMD) is classified as a third-generation sulfonylurea derivative used in the treatment of non-dependent type II diabetes. The reduction in blood glucose levels with GMD is attributed to the elevation of insulin levels through stimulation of pancreatic beta cells^1^. Additionally, GMD improves the activity of intracellular insulin receptors^2^. GMD shows pH-dependent solubility with slight solubility in most solvents. Bioavailability fluctuation problems in antidiabetic oral therapy could be related to severe hypoglycaemia and gastrointestinal disturbances. The challenges of low solubility and variable bioavailability have attracted attention to the design of a more efficient dosage formulation for delivery of GMD^1,3–5^.

Effective management of diabetes can be achieved through the development of a GMD-loaded sustained-release formula. Previous studies in our laboratory investigated the development of sustained-release formulations of GMD. These studies include the development of the triblock *in situ* gel (ISG) implant and self-nanoemulsifying, liposomal and ethosomal transdermal films^6–9^. *In situ* gel (ISG) formulations with biodegradable polymers are one of the applied methods for sustained delivery of drugs. The use of an effective ISG formulation improves patient compliance and sustains GMD action with a consequently reduced frequency of dosing. ISG formulations rely on a solution of the polymer poly (d, l-lactide-co-glycolide) (PLGA) dissolved in a biocompatible and water-miscible or partially miscible solvent. The main challenge in ISG formulation design is the initially elevated amount of the drug released during the sol/gel transformation process of the polymer in the ISG formulation after injection^10^. The initial burst effect can result in exceeding the minimum toxic plasma concentration of the administered drug because of the increased amount of drug load (10–20 times the regular daily dose) included in the formula to cover the long period of release. The strategies used to reduce the initial burst effect include the incorporation of plasticizers such as polyethylene glycol (PEG) and the use of a hydrophobic solvent^11–13^.

As such, a convenient depot of safe and effective antidiabetic therapy is the goal of this study. The Box-Behnken experimental design is used to investigate the factors that affect the performance of the GMD-ISG formula in the management of insulin non-dependent type II diabetes. Different formulations were prepared per the Box-Behnken experimental design. The studied factors were PLGA % (X_1_), PEG % (X_2_), and benzyl benzoate (BB) % in an N-methyl-2-pyrrolidone (NMP) solvent (X_3_). Characterization of the prepared formulations was conducted to achieve the best performing formula. In addition, the *in vivo* pharmacokinetic parameters and hypoglycaemic activities were investigated in an animal model. This approach to controlling the release of GMD using *in situ* based biodegradable polymers could be useful for improving patient compliance and diabetes treatment effectiveness.

## Results and Discussion

The major obstacle in the design of ISG formulations is the high initial burst of the drug after administration^6,13–16^. Accordingly, the goal was to sustain the release of GMD from the ISG formulation by controlling its initial burst from the designed formula. To attain this goal, the Box-Behnken design was applied for multiple response optimization of the data obtained from fifteen prepared formulations of GMD-ISG evaluated for drug release. The selected three factors, with three levels that primarily affect the reduction of GMD initial burst and sustain its release over a longer period, were considered in this study.

### Surface morphology examination using SEM

**Figure 1:**
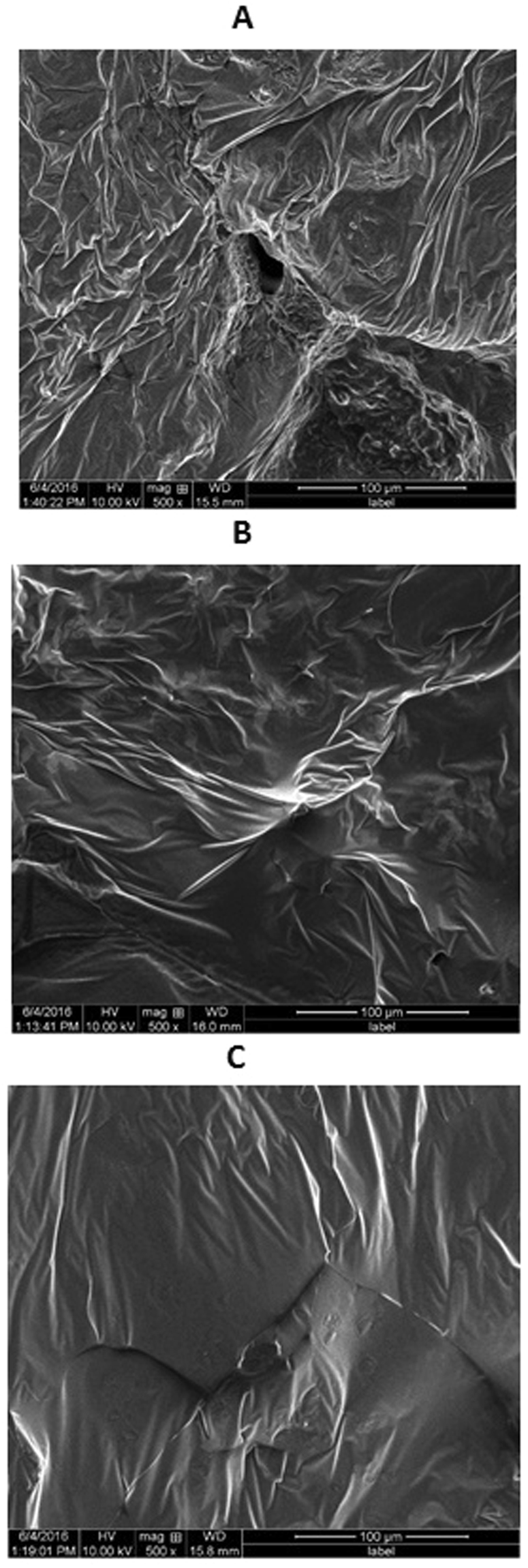
SEM Photographs showing the effect of increasing concentrations of PEG on the surface of GMD-ISG systems. (**A**) 0% PEG, (**B**) 5% PEG, (**C**) 10% PEG.

SEM photographs (Fig. 1) revealed that an increase in the percentage of PEG improved the smoothness and reduced the porosity of the prepared ISG formulation surfaces. These changes in the formulation surface characteristics lead to a reduction in the surface area exposed to the dissolution media, which subsequently reduces the initial burst effect of GMD release.

### ***In vitro*** release of GMD from ISG formulations

**Figure 2:**
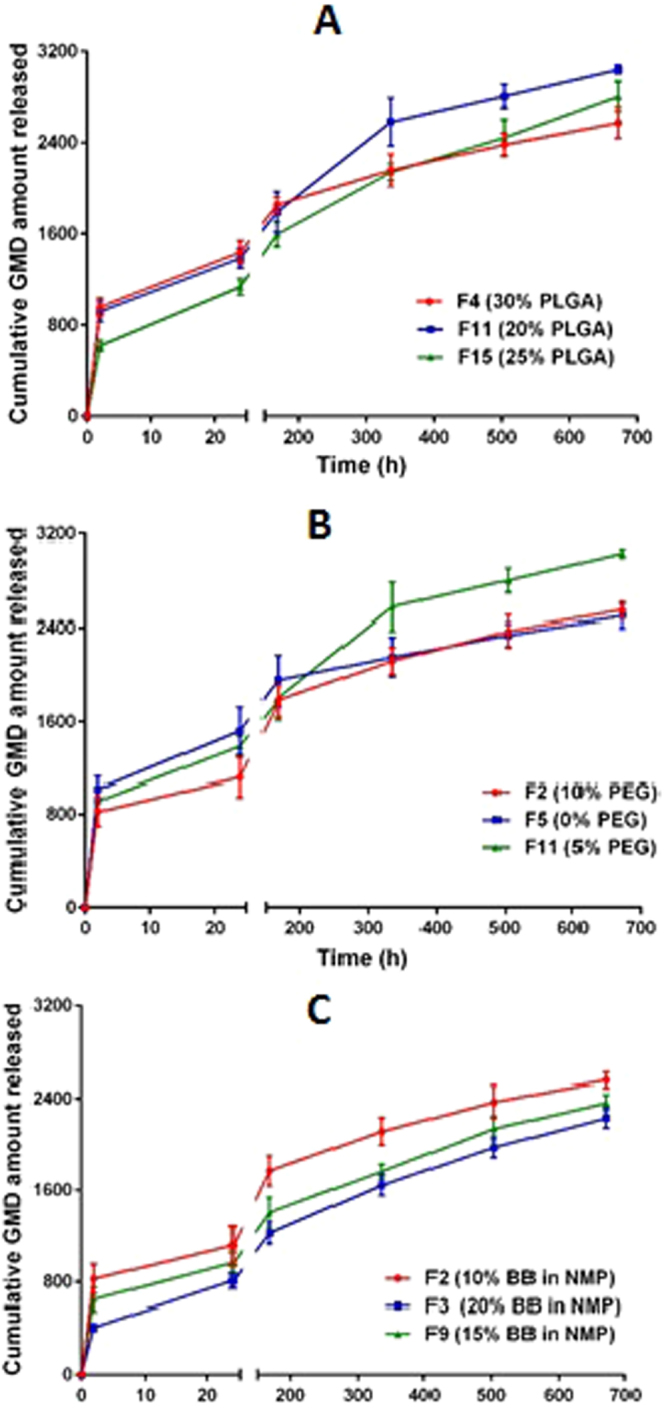
Influence of independent factors on the release profile of GMD from ISG formulations: (**A**) PLGA % (X_1_); (**B**) PEG % (X_2_); (**C**) BB % in NMP (X_3_).

The initial amount of GMD released after 2 and 24 h and the cumulative amount released after 28 days are presented in Table 1. The effect of increasing the concentration of each studied factor on the release profile is presented in Fig. 2. The results show that the maximum amount of GMD released after 28 days (3039.05 µg, 100%) was from the F11 formula, which contains low values of both X_1_ andX_3_. Formula F14, which contains high levels of both X_1_ and X_2_, released the minimum amount of GMD (1803.92 µg, 60.1%). However, F3, which contains high levels of both X_2_ and X_3_, reduced the initial burst of GMD to 412.03 µg (13.7%) and 806.46 µg (26.9%) after 2 and 24 h, respectively. In addition, the results revealed that X_1_ plays an important role in decreasing the initial burst of GMD from the prepared formulations. Formula F7, which contains a high level of PLGA, is an example of the previous finding.

**Table 1:** Composition of GMD-ISG formulations, their independent variables and observed dependent variables.

Formulation code	Independent variables			Observed values of dependent variables		
	X_1_	X_2_	X_3_	Y_1_	Y_2_	Y_3_
F1	25	5	15	608.0	1111.3	2787.25
F2	25	10	10	823.58	1114.11	2551.58
F3	25	10	20	412.03	806.46	2230.13
F4	30	5	10	956.57	1438.05	2570.78
F5	25	0	10	1020.24	1520.53	2498.9
F6	20	5	20	651.55	959.69	2972.38
F7	30	5	20	436.37	904.72	2517.45
F8	25	5	15	641.66	1157.96	2807.25
F9	20	10	15	647.48	1162.63	2561.7
F10	30	0	15	745.37	1203.13	2181.8
F11	20	5	10	918.22	1386.36	3039.05
F12	20	0	15	890.82	1399.3	2928.37
F13	25	0	20	466.91	919.2	2965.57
F14	30	10	15	578.7	1134.63	1803.92
F15	25	5	15	618.0	1134.63	2803.92

Note: *The observed values of Y_1_, Y_2_ and Y_3_ represent the means of three determinations; standard deviations were < 5% of the mean and thus are omitted from the table.

Abbreviations: PLGA, poly (D, L-lactide-co-glycolide); PEG, polyethylene glycol; X_1_, PLGA %; X_2_, PEG %; X_3_, BB % in NMP; Y_1_, initial amount of GMD released after 2 hours (μg); Y_2_, amount of GMD released after 24 hours (μg); Y_3_, cumulative amount of GMD released after 28 days (μg).

### Optimization of GMD-ISG using the response surface methodology

To reduce the rate of GMD release, the main factors that affect the release were investigated using the Box-Behnken experimental design. The mathematical design was applied to investigate, optimize, and explore the main interactions and quadratic effects of the selected factors on the initial burst after 2 and 24 h and the cumulative amount of GMD released after 28 days from the ISG formulations. These main factors were PLGA % (X_1_), PEG % (X_2_) and the percent of BB in NMP (X_3_). These factors were selected based on previous and preliminary studies conducted in our laboratory.

### Assessment of the quantitative effects of the factors

Statgraphics^®^ software was used to statistically analyse the 15 batch results from the Box-Behnken experimental design with two-way ANOVA followed by multiple regression analysis. Table 2 shows the estimated effects of the factors, the *F*-ratios, and the associated *p*-values on the three responses. A *p*-value of less than 0.05 is considered significant.

**Table 2:** Estimated effects of factors, *F*-ratios, and associated *P-*values for all responses (Y_1_, Y_2_, and Y_3_).

Factor	Y_1_			Y_2_			Y_3_		
	Estimate	*F*-ratio	*P*-values	Estimate	*F*-ratio	*P*-values	Estimate	*F*-ratio	*P*-values
X_1_	−97.77	12.14	0.0176*	−170.52	2.44	0.1788	−606.89	71.22	0.0004*
X_2_	−165.39	34.75	0.0020*	−108.34	32.08	0.0024*	−356.83	24.62	0.0042*
X_3_	−437.94	243.62	0.0001*	23.62	164.93	0.0001*	6.31	0.01	0.9335
X_1_^2^	153.03	13.73	0.0139*	3.45	10.37	0.0235*	−217.16	4.21	0.0955
X_1_ X_2_	38.34	0.93	0.3784	1.68	2.67	0.1631	−5.61	0.00	0.9582
X_1_ X_3_	−126.77	10.21	0.0241*	−1.07	1.07	0.3475	6.67	0.00	0.9503
X_2_^2^	33.05	0.64	0.4599	0.16	0.02	0.8848	−643.9	37.00	0.0017*
X_2_ X_3_	70.89	3.19	0.1341	2.94	8.14	0.0357*	−394.06	15.01	0.0117*
X_3_^2^	83.22	4.06	0.1000	−1.95	3.30	0.1290	168.04	2.52	0.1733

Note: *Significant effect of factors on individual dependent variables.

Abbreviations: X_1_, PLGA %; X_2_, PEG %; X_3_, BB % in NMP; Y_1_, initial amount of GMD released after 2 hours (μg); Y_2_, amount of GMD released after 24 hours (μg); Y_3_, cumulative amount of GMD released after 28 days (μg); ${{\rm{X}}}_{1}^{2}$, ${{\rm{X}}}_{2}^{2}$, and ${{\rm{X}}}_{3}^{2}$ are the quadratic terms for the factors, and X_1_ X_2_, X_1_ X_3_, and X_2_ X_3_ are the interaction terms between the factors.

**Figure 3:**
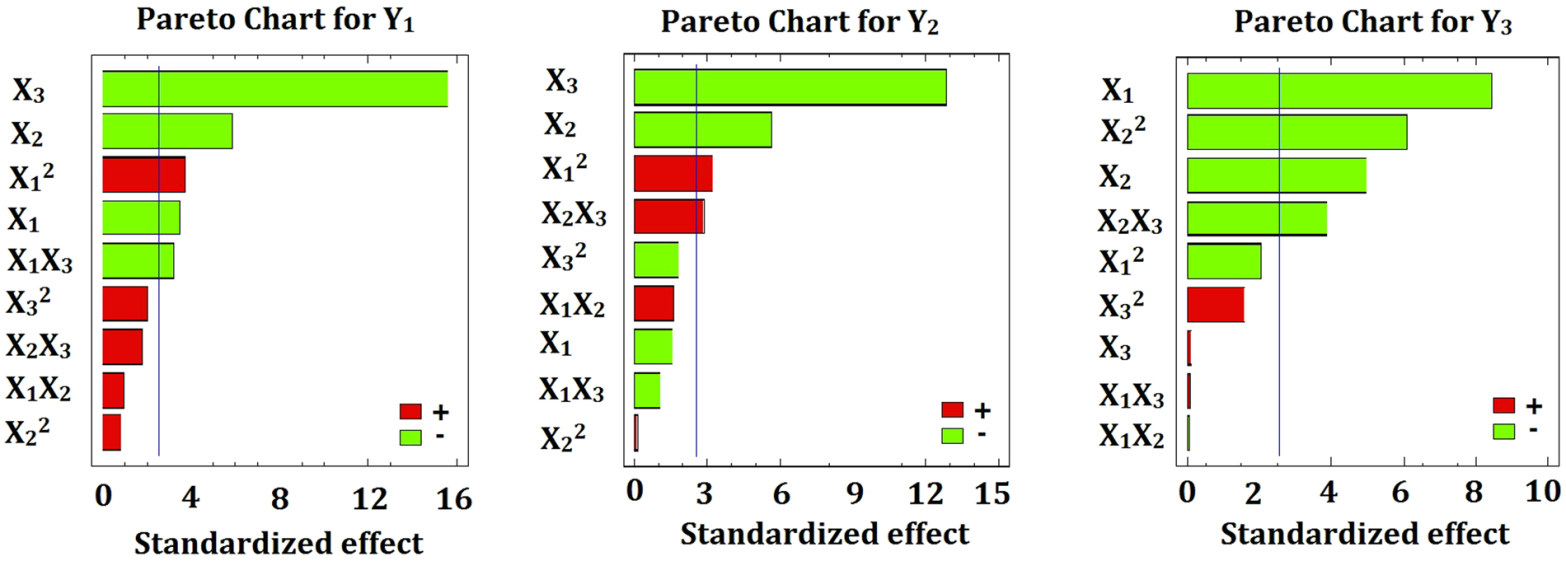
Standard Pareto charts showing the effects of X_1_, X_2_ and X_3_ and their combined effects on Y_1_-Y_3_.

According to the Pareto chart in Fig. 3 and the results in Table 2, the percentage of PEG (X_2_) is the only factor that showed significant effects on the three investigated responses, Y_1_, Y_2_, and Y_3_, with *p*-values of 0.002, 0.0024, and 0.0042, respectively. The percentage of PLGA (X_1_) has a significant antagonistic effect on the initial burst after 2 h (Y_1_) and the cumulative amount released after 28 days (Y_3_) with *p*-values of 0.0176 and 0.0004, respectively. The percentage of BB (X_3_) significantly affects the initial burst after 2 h (Y_1_) and after 24 h (Y_2_) with the same *p*-value of 0.0001. In addition, it was found that the quadratic term of X_1_ significantly affects the initial burst responses, Y_1_ and Y_2_, with *p*-values of 0.0139 and 0.0235, respectively. The quadratic term of X_2_ has a significant effect on the cumulative amount of GMD released after 28 days (Y_3_) with a *p*-value of 0.0017. The interaction of X_1_X_3_ significantly affects the initial burst after 2 h with a *p*-value of 0.0241. The interaction of X_2_X_3_ significantly affects the release after 24 h and 28 days with *p*-values of 0.0357 and 0.0117, respectively.

### Statistical analysis and mathematical modelling of the experimental data

The response values released for the initial burst after 2 h (Y_1_), the initial burst after 24 h (Y_2_) and the cumulative release after 28 days (Y_3_) were analysed. A mathematical model for each response was created, and these models are presented in Equations 1–3.

1 $$\begin{array}{ccc}{\rm{I}}{\rm{n}}{\rm{i}}{\rm{t}}{\rm{i}}{\rm{a}}{\rm{l}}\,{\rm{a}}{\rm{m}}{\rm{o}}{\rm{u}}{\rm{n}}{\rm{t}}\,{\rm{o}}{\rm{f}}\,{\rm{G}}{\rm{M}}{\rm{D}}\,{\rm{r}}{\rm{e}}{\rm{l}}{\rm{e}}{\rm{a}}{\rm{s}}{\rm{e}}{\rm{d}}\,{\rm{a}}{\rm{f}}{\rm{t}}{\rm{e}}{\rm{r}}\,2\,{\rm{h}}{\rm{o}}{\rm{u}}{\rm{r}}{\rm{s}}\,({{\rm{Y}}}_{1}) & = & 3161.86-128.607\,{{\rm{X}}}_{1}\\  &  & -\,63.584\,{{\rm{X}}}_{2}-37.433\,{{\rm{X}}}_{3}\\  &  & +\,3.061\,{{\rm{X}}}_{1}^{2}+0.767\,{{\rm{X}}}_{1}{{\rm{X}}}_{2}\\  &  & -\,2.535\,{{\rm{X}}}_{1}{{\rm{X}}}_{3}+0.661\,{{\rm{X}}}_{2}^{2}\\  &  & +\,1.418\,{{\rm{X}}}_{2}{{\rm{X}}}_{3}+1.664\,{{\rm{X}}}_{3}^{2}\end{array}$$

2 $$\begin{array}{ccc}{\rm{A}}{\rm{m}}{\rm{o}}{\rm{u}}{\rm{n}}{\rm{t}}\,{\rm{o}}{\rm{f}}\,{\rm{G}}{\rm{M}}{\rm{D}}\,{\rm{r}}{\rm{e}}{\rm{l}}{\rm{e}}{\rm{a}}{\rm{s}}{\rm{e}}{\rm{d}}\,{\rm{a}}{\rm{f}}{\rm{t}}{\rm{e}}{\rm{r}}\,24\,{\rm{h}}{\rm{o}}{\rm{u}}{\rm{r}}{\rm{s}}\,({{\rm{Y}}}_{2}) & = & 3832.83-170.518\,{{\rm{X}}}_{1}\\  &  & -\,108.335\,{{\rm{X}}}_{2}+23.62\,{{\rm{X}}}_{3}\\  &  & +\,3.448\,{{\rm{X}}}_{1}^{2}+1.682\,{{\rm{X}}}_{1}{{\rm{X}}}_{2}\\  &  & -\,1.067\,{{\rm{X}}}_{1}{{\rm{X}}}_{3}+0.163\,{{\rm{X}}}_{2}^{2}\\  &  & +\,2.937\,{{\rm{X}}}_{2}{{\rm{X}}}_{3}-1.945\,{{\rm{X}}}_{3}^{2}\end{array}$$

3 $$\begin{array}{rcl}{\rm{Cumulative}}\,{\rm{amount}}\,{\rm{of}}\,{\rm{GMD}}\,{\rm{released}}\,{\rm{after}}\,28\,{\rm{days}}\,({{\rm{Y}}}_{3}) & = & 1650.35+155.027\,{{\rm{X}}}_{1}\\  &  & +\,214.117\,{{\rm{X}}}_{2}-64.122\,{{\rm{X}}}_{3}\\  &  & -\,4.343\,{{\rm{X}}}_{1}^{2}-0.112\,{{\rm{X}}}_{1}{{\rm{X}}}_{2}\\  &  & +\,0.133\,{{\rm{X}}}_{1}{{\rm{X}}}_{3}-12.878\,{{\rm{X}}}_{2}^{2}\\  &  & -\,7.881\,{{\rm{X}}}_{2}{{\rm{X}}}_{3}+3.361\,{{\rm{X}}}_{3}^{2}\end{array}$$

Equations 1–3 reflect the quantitative effects of the factors on the initial burst of GMD release after 2 h (Y_1_), the amount of GMD released after 24 hours (Y_2_) and the cumulative amount of GMD released after 28 days (Y_3_). Plotting of Pareto charts determines the effect of the investigated factors, their interactions, and their quadratic effects on the dependent responses (Fig. 3). It was found that PEG % (X_2_) has a relatively larger coefficient in the regression equations, which endorses its significant effect on the studied responses because its bar extends beyond the reference line. As shown in Fig. 3 the percentage of PLGA (X_1_) has an antagonistic effect on the initial burst after 2 h (Y_1_) and the cumulative amount released after 28 days (Y_3_). The percentage of BB in NMP (X_3_) displayed an antagonistic effect on the initial burst after 2 h (Y_1_) and the amount released after 24 h (Y_2_), whereas the quadratic term of the factor X_1_ had a synergistic effect on the same responses.

**Figure 4:**
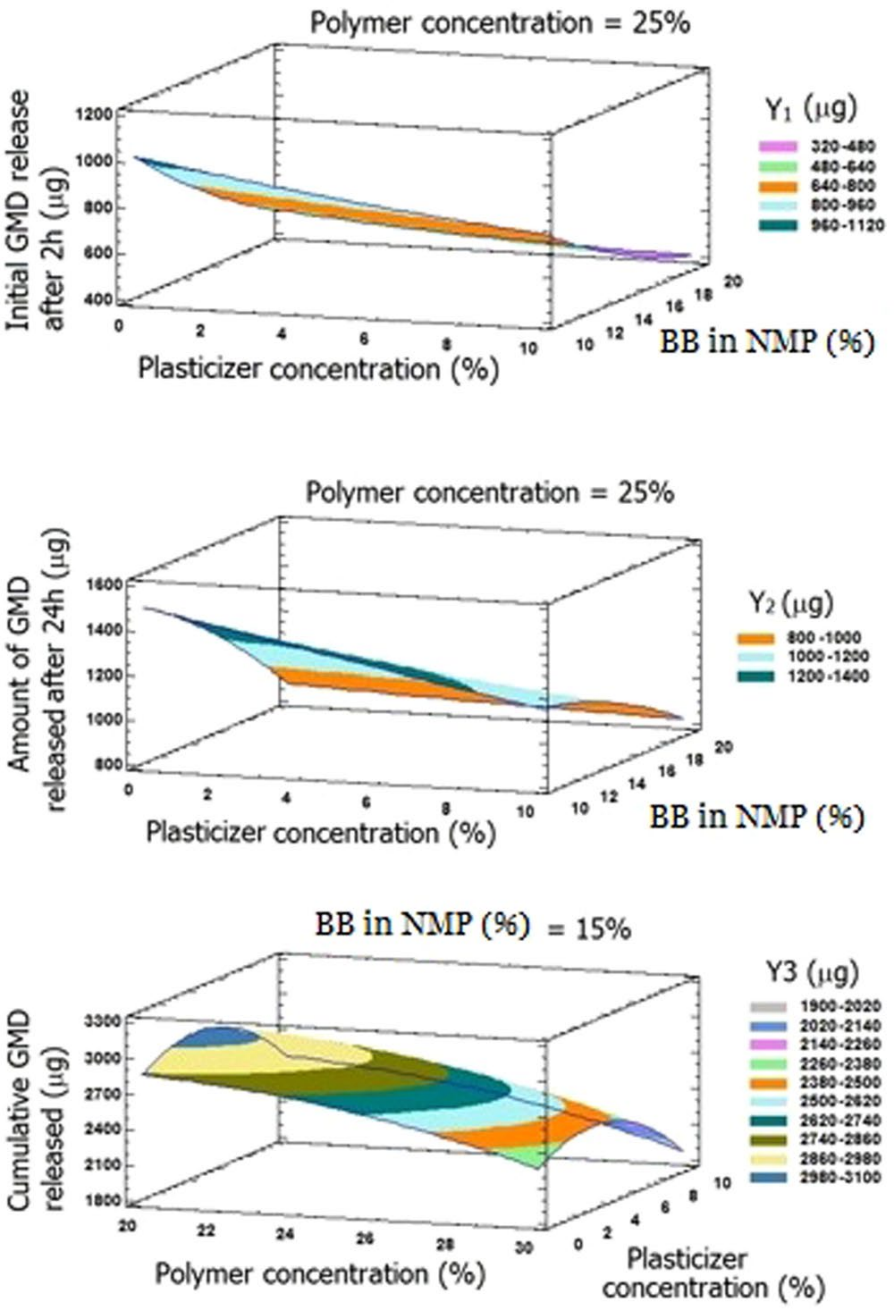
3D response surface plots showing the effects of X_1_, X_2_ and X_3_ on the investigated responses Y_1_ (top), Y_2_ (middle) and Y_3_ (bottom).

It was noted from the 3D response surface plot (Fig. 4) that an inverse relationship exists between X_1_ and Y_3_, which indicates that the PLGA % (X_1_) determines the cumulative amount of GMD released after 28 days (Y_3_). At the same concentrations of both X_2_ and X_3_, as the concentration of PLGA increased from 20 to 30%, Y_3_ decreased from 2972.38 in F6 to 2517.45 µg in F7, from 2928.37 in F12 to 2181.8 µg in F10, and from 3039.05 in F11 to 2570.78 µg in F4. This observation can be attributed to the presence of PLGA at higher concentrations, which hastens the solidification of the formulation and subsequently reduces the leaching of GMD to the surrounding phase^6,15,17,18^. To a certain extent, the same finding was attained in the initial burst after 2 h in the previous formulations. However, incorporation of PEG in the ISG formulation significantly affects all of the release parameters investigated in this study. The Pareto charts (Fig. 3) and 3D response surface plots (Fig. 4) demonstrate that an inverse relationship exists between the PEG % (X_2_) and the release of GMD from ISG at all time points of the study. When the PEG concentration increased from 0 to 10% in the formulation with constant levels of X_1_ and X_3_, the initial burst of GMD after 2 h (Y_1_) decreased from 1020.24 to 823.58 µg in F5 and F2, respectively; from 890.82 to 647.48 µg in F12 and F9, respectively; and from 745.37 to 578.7 µg in F10 and F14, respectively. Additionally, the amount of GMD released after 24 h (Y_2_) decreased as the PEG concentration in the formulations increased from 0 to 10%. Similarly, Y_2_ decreased from 1520.53 to 1114.11 µg in F5 and F2, respectively; from 1399.3 to 1162.63 µg in F12 and F9, respectively; and from 1203.13 to 1134.63 µg in F10 and F14, respectively. Finally, the increase in PEG concentration in the ISG formulations decreased the cumulative amount of GMD released after 28 days, which confirms the sustained release pattern of GMD from ISG formulations. This postulation was verified from the obtained results in which Y_3_ increased from 1803.92 in F14 to 2181.8 µg in F10 by decreasing the percent of PEG (X_2_) from 10% to 0% at the same level of X_1_ and X_3_. This result could be attributed to the decrease in the transition temperature of PLGA, which leads to rapid congealing of the formulation as a result of the increase in the percentage of PEG in the formulation^12^. In addition, SEM photographs (Fig. 1) revealed that an increase in the percentage of PEG improved the smoothness and reduced the porosity of the ISG formulation surface, which decreased the surface area exposed to the dissolution media and subsequently reduced the release of GMD^12,19^.

Finally, the release behaviour of GMD from the ISG formulations was affected by the hydrophobicity of the solvent from which they were prepared, i.e., the percentage of BB in NMP (X_3_). It was noted that X_3_ significantly affected the initial burst after 2 h (Y_1_) and the amount released after 24 h (Y_2_). In contrast, X_3_ did not show a significant effect on the cumulative amount of GMD released after 28 days (Y_3_), and the Y_1_ and Y_2_ values decreased with the increase in the percentage of BB from 10 to 20%. The amount of GMD released was 823.58 µg in F2 and decreased to 412.03 µg in F3 after 2 h. In addition, the amount of GMD released was 1114.11 µg in F2 and decreased to 806.46 µg in F3 after 2 h. This finding can be attributed to the effect of BB in decreasing the solvent affinity of PLGA solutions for aqueous fluids to slow the rate of phase inversion and yield a more uniform prolonged release^15,20–22^.

### Prediction of the optimized GMD-ISG formulation

After analysis of the experimental factors, an optimum combination of these factors was achieved. The results of the analysis suggested an optimized ISG formulation containing 25.4% PLGA, 3.4% PEG, and 20% BB in NMP. The optimized formula was prepared and evaluated to confirm the validity of the observed optimal parameters and the predicted responses. The observed values for Y_1,_ Y_2_, and Y_3_ were 476.42 µg (15.9%), 877.64 µg (29.2%) and 3105.85 µg (103.5%), respectively, and the predicted values were 454.102, 857.893 and 2952.38 µg, respectively. As a result, it can be concluded that the optimized combination of the independent factors confirmed the desired release behaviour of GMD from the ISG formulations. This outcome also verified the reliability of the optimization procedure in the development of a GMD-ISG formulation characterized by a sustained release pattern.

### ***In vivo*** study

#### Assessment of the GMD-ISG hypoglycaemic efficacy

**Figure 5:**
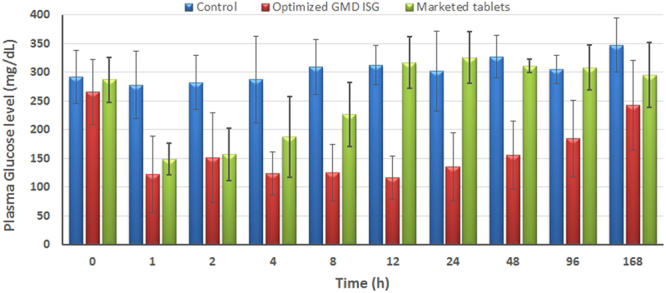
Hypoglycaemic activity of the administered GMD from an optimized GMD-ISG formula (dose 10 mg/kg, IM injection) and marketed oral tablets (dose 1 mg/kg, oral).

The hypoglycaemic efficacies of the optimized GMD-ISG formula and marketed tablets showed a reduction in plasma glucose levels compared with the control group after 1, 2 and 4 h of dose administration (Fig. 5). The optimized GMD-ISG formula extended the reduction in glucose levels compared with the control group for up to 168 h after administration. These results showed the effectiveness of the depot action of the optimized GMD-ISG formulation in reducing glucose levels for a longer period than the marketed GMD tablets.

#### Quantification of the GMD plasma levels

**Figure 6:**
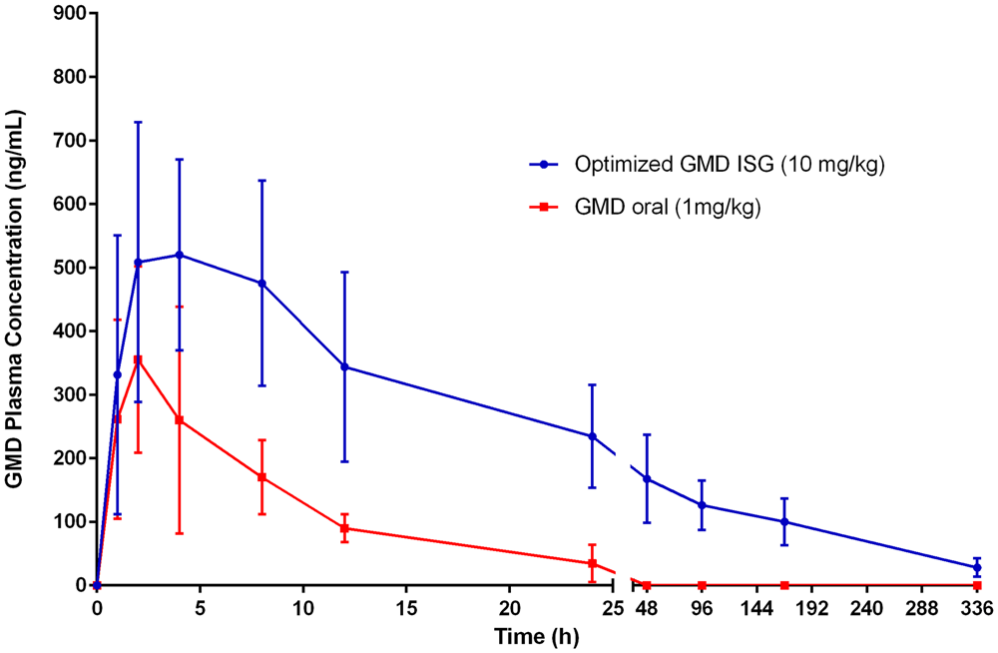
Means of GMD plasma concentration-time profiles for an optimized GMD-ISG formula (10 mg/kg, IM injection) and marketed GMD tablets (1 mg/kg, oral).

The initial burst is a major concern in the design and development of depot ISG formulations. The goal of this investigation was to estimate the GMD plasma levels of the optimized GMD-ISG formula compared with those of oral administration of marketed GMD tablets to assess the initial burst effect and the C_max_ levels of GMD. The injected optimized depot GMD dose was 10 times the administered oral dose. The results in Fig. 6 revealed no significant difference (p < 0.05) in C_max_ values between the optimized ISG and the marketed oral dose formulations. The optimized GMD-ISG formula showed a C_max_ of 520.168 ± 168.642 ng/mL, whereas the marketed oral tablets showed a C_max_ of 355.213 ± 144.998 ng/mL. Additionally, the optimized GMD-ISG formula and marketed oral tablets showed t_max_ values of 4 h and 2 h, respectively. These results revealed that the initial burst of the optimized depot GMD-ISG formula did not significantly exceed the C_max_ of the marketed oral (low-dose) tablets. The inclusion of the optimum levels of factors (X_1_, X_2_, and X_3_ parameters) from the experimental design in the formulation reduced the initial amount of GMD released after injection of the GMD-ISG formula. Accordingly, these results indicated the possibility of safe administration for the optimized depot GMD-ISG dose.

Achievement of long-lasting GMD therapy could have a positive impact on the antidiabetic therapy^23^. Depot treatment could improve patient compliance and reduce the individual variation in bioavailability that could result from GMD oral treatment. In addition, depot therapy could improve the correlation between the administered GMD dose and its plasma concentration compared with oral therapy. Furthermore, the control of plasma glucose levels and reduction of side effects are better achieved when adjusting the dose frequency of the depot ISG formula, and the prescriber can be confident that the patient receives the required dose. However, findings should be examined in further pre/clinical studies that are necessary prior to the use of the GMD-ISG formula as a valuable alternative to oral antidiabetic therapy for patient convenience and compliance with minimum or no risks of inadvertent hypoglycaemia.

## Materials and Methods

### Materials

GMD was a kind gift from Spimaco Addwaeih, Riyadh, KSA. Poly(DL-lactide-co-glycolide) (PLGA, 50:50, inherent viscosity range 0.55–0.75 dL/g) was sourced from LACTEL Absorbable polymers, Birmingham, AL, USA. Polyethylene glycol 200 (PEG 200), benzyl benzoate (BB) and N-methyl-2-pyrrolidone (NMP) were purchased from Sigma Aldrich Corporate (St. Louis, MO, USA).

### Box-Behnken experimental design

Three factors were selected per the obtained preliminary data. These factors were PLGA % (X_1_), PEG % (X_2_), and BB % in NMP (X_3_). Their effects on the initial amount of GMD released after 2 h (Y_1_), the amount of GMD released after 24 h (Y_2_) and the cumulative amount of GMD released after 28 days (Y_3_) were investigated. The development of different GMD-ISG formulations per various levels of independent variables (Table 3) was conducted using the Box-Behnken experimental design (Statgraphics Centurion XV version 15.2.05 software, StatPoint Technologies Inc., Warrenton, VA, USA). The experimental design was chosen to minimize all of the investigated dependent variables. The polynomial equations generated from the obtained results were used to obtain the relationships between the investigated independent variables and the dependent variables.

**Table 3:** Independent and dependent variables in the Box-Behnken experimental design.

Independent variables	Levels		
PLGA %, (X_1_)	20	30	40
PEG %, (X_2_)	0	5	10
BB % in NMP, (X_3_)	10	15	20
**Dependent variables**	**Constraints**		
	**Low**	**High**	**Target**
Initial burst after 2 hours, % (Y_1_)	412.03	1020.24	Minimize
Initial burst after 24 hours, % (Y_2_)	806.46	1520.53	Minimize
Cumulative release after 28 days, % (Y_3_)	1803.92	3039.05	Maximize

Abbreviations: PLGA, poly (D, L-lactide-co-glycolide); PEG, polyethylene glycol; BB, benzyl benzoate; NMP, N-methyl-2-pyrrolidone.

### Formulation of GMD loaded ***in situ*** gel formulations

The specified amount of polymer PLGA was dissolved in 2 mL of the solvent system per the Box-Behnken experimental design. GMD (6 mg) was added to the polymeric solution and mixed by vortexing until complete dissolution. All prepared formulations were injected through a 21-gauge needle.

### Surface morphology examination using scanning electron microscopy

The surface characteristics of the optimized GMD formulations were investigated after gelation and lyophilization using scanning electron microscopy (SEM). For the gelation process, each formulation was injected separately into a buffer solution of pH 7.4 at 37 °C, held for 24 h, and finally collected and lyophilized. The lyophilized formulation was mounted on metal stubs with conductive silver paint, sputtered with gold and subjected to characterization using SEM (Philips XL30; FEI, Hillsboro, OR, USA). The resulting photographs were used to investigate the effect of PEG concentrations (0%, 5% and 10% PEG) on the surface morphology of the selected GMD-ISG systems.

### ***In vitro*** release of GMD biodegradable ISG formulations

*In vitro* GMD release from the ISG formulations was investigated via the modified dialysis method, as described previously^6^. In brief, a GMD formula equivalent to 3 mg was injected into a dialysis tube (Sigma-Aldrich, St. Louis, MO. USA, molecular weight cut-off of 12,000 Da) containing 10 mL of phosphate buffered solution at pH 7.2. After sealing, the tubes were inserted into vessels of the USP II dissolution apparatus containing 250 mL of buffer at pH 7.2 and 37 °C with agitation at 100 rpm. Aliquots of 5 mL each were assayed for GMD content using HPLC, as described in the *in vivo* experimental section. Experiments were performed in triplicate, and the mean cumulative GMD amount released ± S.D. was calculated.

### ***In vivo*** study

#### Assessment of the GMD-ISG hypoglycaemic efficacy

To evaluate the hypoglycaemic activity of GMD, male Wistar rats weighing 200–250 g were used in this study. The animals were supplied by King Fahd Medical Research Centre, Jeddah, Saudi Arabia. Animal use was conducted per the Helsinki agreement protocol, the Guiding Principle in Care and Use of Animals (DHEW publication NIH 80–23), and the requirements and the approval of the Research Ethics Committee, Faculty of Pharmacy, King Abdulaziz University, Jeddah, Saudi Arabia.

Diabetes was induced by intraperitoneal injection with 50 mg/kg streptozotocin two weeks prior to the study^8^. Fasting blood glucose levels were assessed using Accu-Chek^®^ Go (Roche, Mannheim, Germany). Rats with moderate diabetes, i.e., fasting blood glucose levels in the range of 250–350 mg/dl, were selected for the study.

The animals were divided into 3 groups. The first group was injected intramuscularly with 250 μL of plain ISG formula representing the negative control. Group 2 was given commercial GMD tablets orally at 1 mg/kg body weight^24^. Group 3 was injected intramuscularly with 250 μL of the optimized GMD-ISG formula (equivalent to 10 mg GMD).

#### Quantification of the GMD plasma levels

Pharmacokinetic calculations based on plasma GMD concentrations were performed via non-compartmental analysis using groups 2 and 3, as described in the previous section. The maximum plasma concentration (C_max_) and time point of maximum plasma concentration (t_max_) were calculated using Kinetica software v4.2 (Thermo Scientific Corporation, Philadelphia, PA, USA). Blood samples (0.25 mL) were withdrawn at 0 (pre-dose), 1, 2, 4, 8, 12, 24, 48, 96, 168 and 336 hours. GMD concentrations were analysed using the HPLC method. The HPLC system consisted of an Agilent 1200 system, a solvent delivery module, a quaternary pump, an autosampler, a diode-array detector (DAD), and a column compartment (Agilent Technologies, Santa Clara, CA, USA). The separation was performed on an Agilent Zorbax Eclipse Plus C18 column, 3.5 µm, 4.6 × 100 mm and maintained at 35 °C. The analytes were isocratically eluted using a mobile system composed of acetonitrile:0.1% formic acid in water (60:40, v/v) and pumped at a flow rate of 1 mL/min with detection at λ  = 230 nm. Plasma GMD concentrations were analysed after liquid-liquid extraction with methanol using the Sujatha *et al*. method^25^.

### Data availability

The datasets generated and/or analysed during the current study are available from the corresponding author on reasonable request.

## Conclusions

The results in this work indicated the successful application of the experimental design to develop a GMD-ISG formulation for long-lasting GMD release. To reduce the rate of GMD release, the main factors that affect the release were investigated via the Box-Behnken experimental design. The results revealed that PEG is the only factor investigated that showed a significant effect on all of the investigated responses. The *in vivo* data from diabetic rats displayed the ability of the optimized formula to reduce plasma glucose levels for extended periods compared with orally administered marketed tablets. The optimized formula dose (10 mg/kg) exhibited no significant difference in the C_max_ of GMD compared with that of the marketed oral tablets dose (1 mg/kg). The achievement of a long-lasting GMD-ISG formula is expected to benefit patient compliance and reduce inadvertent hypoglycaemia.
